# Structure-based prediction of BRAF mutation classes using machine-learning approaches

**DOI:** 10.1038/s41598-022-16556-x

**Published:** 2022-07-22

**Authors:** Fanny S. Krebs, Christian Britschgi, Sylvain Pradervand, Rita Achermann, Petros Tsantoulis, Simon Haefliger, Andreas Wicki, Olivier Michielin, Vincent Zoete

**Affiliations:** 1 Computer-Aided Molecular Engineering Group, Department of Oncology UNIL-CHUV, University of Lausanne, Epalinges, Switzerland; 2 Molecular Modelling Group, SIB Swiss Institute of Bioinformatics, Lausanne, Switzerland; 3 Center for Precision Oncology, Department of Oncology, Centre Hospitalier Universitaire Vaudois, University of Lausanne, Lausanne, Switzerland; 4 Department of Medical Oncology and Hematology, University Hospital Zurich, Comprehensive Cancer Center Zurich, University of Zurich, Zurich, Switzerland; 5 Department of Radiology, Clinic of Radiology and Nuclear Medicine, University Hospital Basel, University of Basel, Basel, Switzerland; 6 Department of Oncology, Hôpitaux Universitaires de Genève, University of Geneva, Geneva, Switzerland; 7 Department of Medical Oncology, Inselspital, Bern University Hospital, University of Bern, Bern, Switzerland

**Keywords:** Cancer genomics, Computational science

## Abstract

The BRAF kinase is attracting a lot of attention in oncology as alterations of its amino acid sequence can constitutively activate the MAP kinase signaling pathway, potentially contributing to the malignant transformation of the cell but at the same time rendering it sensitive to targeted therapy. Several pathologic BRAF variants were grouped in three different classes (I, II and III) based on their effects on the protein activity and pathway. Discerning the class of a BRAF mutation permits to adapt the treatment proposed to the patient. However, this information is lacking new and experimentally uncharacterized BRAF mutations detected in a patient biopsy. To overcome this issue, we developed a new in silico tool based on machine learning approaches to predict the potential class of a BRAF missense variant. As class I only involves missense mutations of Val600, we focused on the mutations of classes II and III, which are more diverse and challenging to predict. Using a logistic regression model and features including structural information, we were able to predict the classes of known mutations with an accuracy of 90%. This new and fast predictive tool will help oncologists to tackle potential pathogenic BRAF mutations and to propose the most appropriate treatment for their patients.

## Introduction

The mitogen-activated protein (MAP) kinase signaling pathway, also called RAS/RAF/MEK/ERK pathway, regulates the cell growth and division via a cascade of kinase phosphorylations^1^. The process is initiated by the binding of an extracellular signaling molecule to the transmembrane kinase receptor EGFR, leading to its dimerization and auto-phosphorylation, and thus, allowing GRB2 interaction, triggering the possibility to form the complex EGFR/GRB2/SOS. Inactive RAS-guanosine-5’-diphosphate (GDP) complex can be activated by SOS from the newly formed complex EGFR/GRB2/SOS, which induces the exchange of GDP to guanosine-5ʹ-triphosphate (GTP). Then, active RAS-GTP will activate BRAF which will be able to form dimers and trigger the kinase cascade involving MEK1/2 and ERK1/2, leading to the activation of cell growth and division.

**Figure 1:**
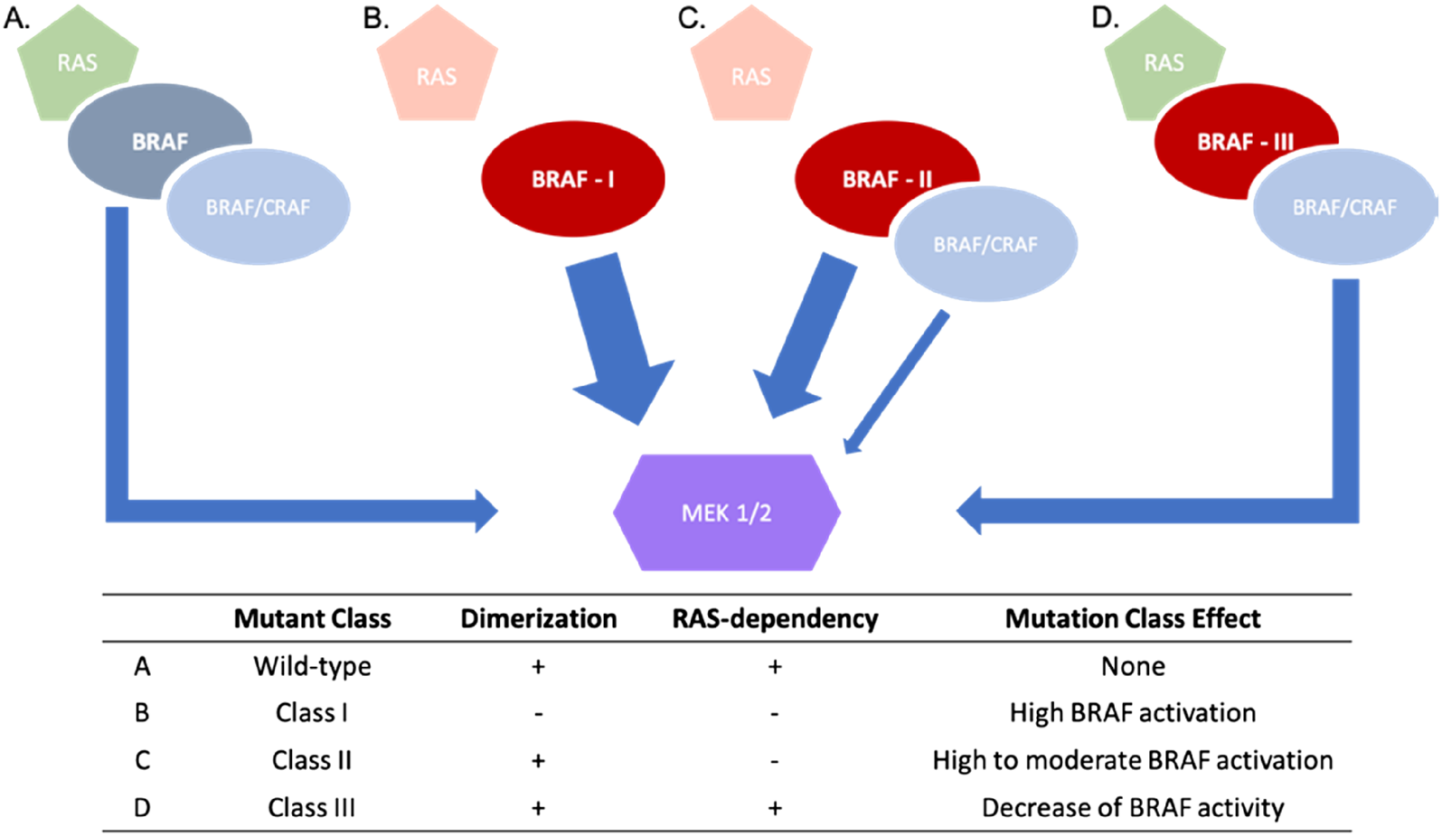
BRAF mutations classes. Schematic description of the wild-type, the three BRAF mutations classes and their functional impacts. The arrow width indicates the potential activation of MEK 1 and 2.

BRAF is therefore a central serine/threonine-protein kinase of the MAP kinase pathway and mutations of this protein can lead to important pathogenic effects. Indeed, several BRAF mutations have been detected in human cancers and characterized as pathogenic, making it an important onco-driver gene^2^. Depending on the position and the amino acid variation, a mutation can have different effects on the functionality of BRAF that could impact treatment possibilities for the patient. Mutations effects analysis has identified three mutation classes^3^ (Fig. 1). Class I mutations lead to a high activation of RAS-independent monomeric BRAF. These variants are all missense mutations of Val600, which resides in the activation loop of the protein. Class II mutations lead to a high and moderate RAS-independent BRAF dimeric activation. The activation induced by these mutations is lower than in class I. Class III mutations are RAS-dependent homo or heterodimers and lead to a decrease or loss of function. Most mutations of these three classes affect the kinase domain of the protein, which is the site of the catalytic activity where the hydrolyzation of the adenosine triphosphate (ATP) into adenosine diphosphate (ADP) occurs to phosphorylate and activate MEK1 or MEK2, and thus, affect the pathway itself.

## Results and discussion

**Figure 2:**
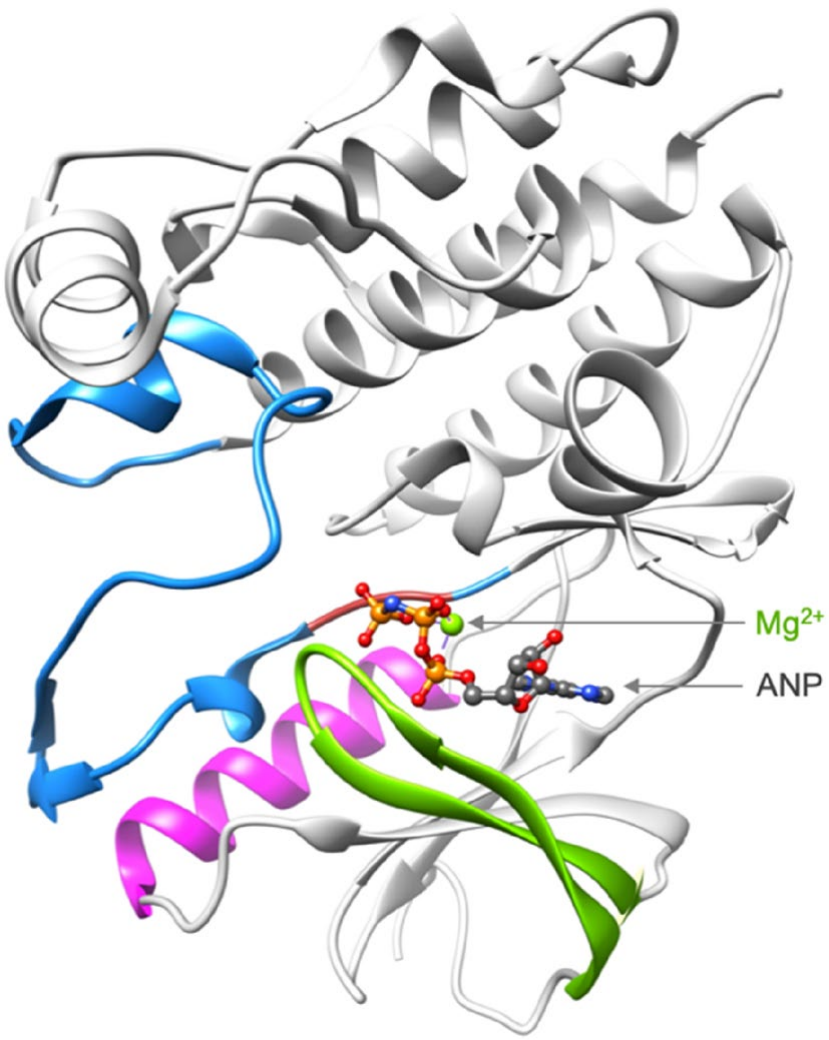
BRAF kinase domain. The key regions of the kinase domain are colored as follows: the P-loop in green, the C-helix in magenta, the activation loop in blue and the kinase DFG motif in crimson. This BRAF structure is extracted from the complex BRAF:MEK (pdb code: 6pp9) ^12^. The BRAF kinase is co-crystallized in the presence of Mg^2+^ and ANP, which is an ATP analog.

The BRAF kinase domain, which extends from amino acid (aa) 457 to 717, contains the catalytic site that allows the phosphorylation of proteins via the hydrolysis of ATP into ADP in presence of the metallic ion Mg^2+^. The domain is composed of three main regions that regulate the kinase activity: (i) the P-loop (aa: 458–475), (ii) the C-helix (aa: 491–506) and (iii) the activation loop (aa: 593–633), which includes the conserved DFG motif (aa: 594–596) (Fig. 2).

**Figure 3:**
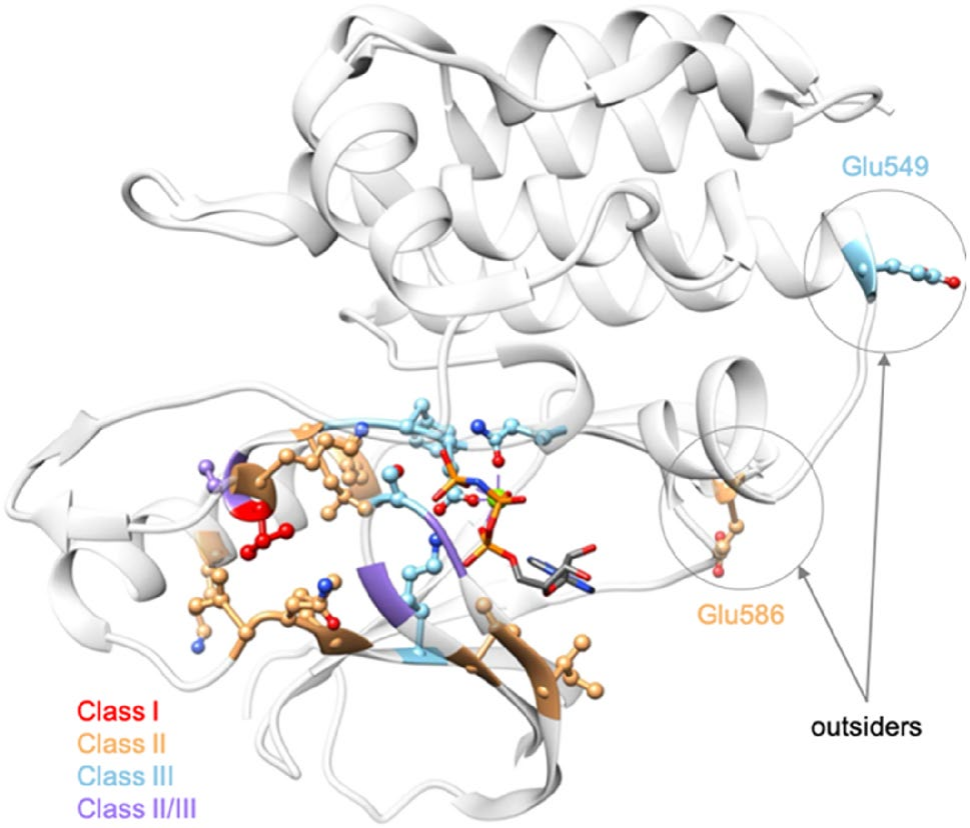
Mapping classes of known mutations in the kinase domain. The residues whose mutation leads to a known mutation class are represented in ball and stick. The one for which the mutation leads to the class I is colored in red, those leading to class II and class III are colored in orange and blue, respectively. The sites of mutations that can be a class II or III depending on the variant are colored in purple. The BRAF kinase is represented in light gray and transparent ribbons to facilitate the visualization (pdb code: 6pp9) ^12^.

**Figure 4:**
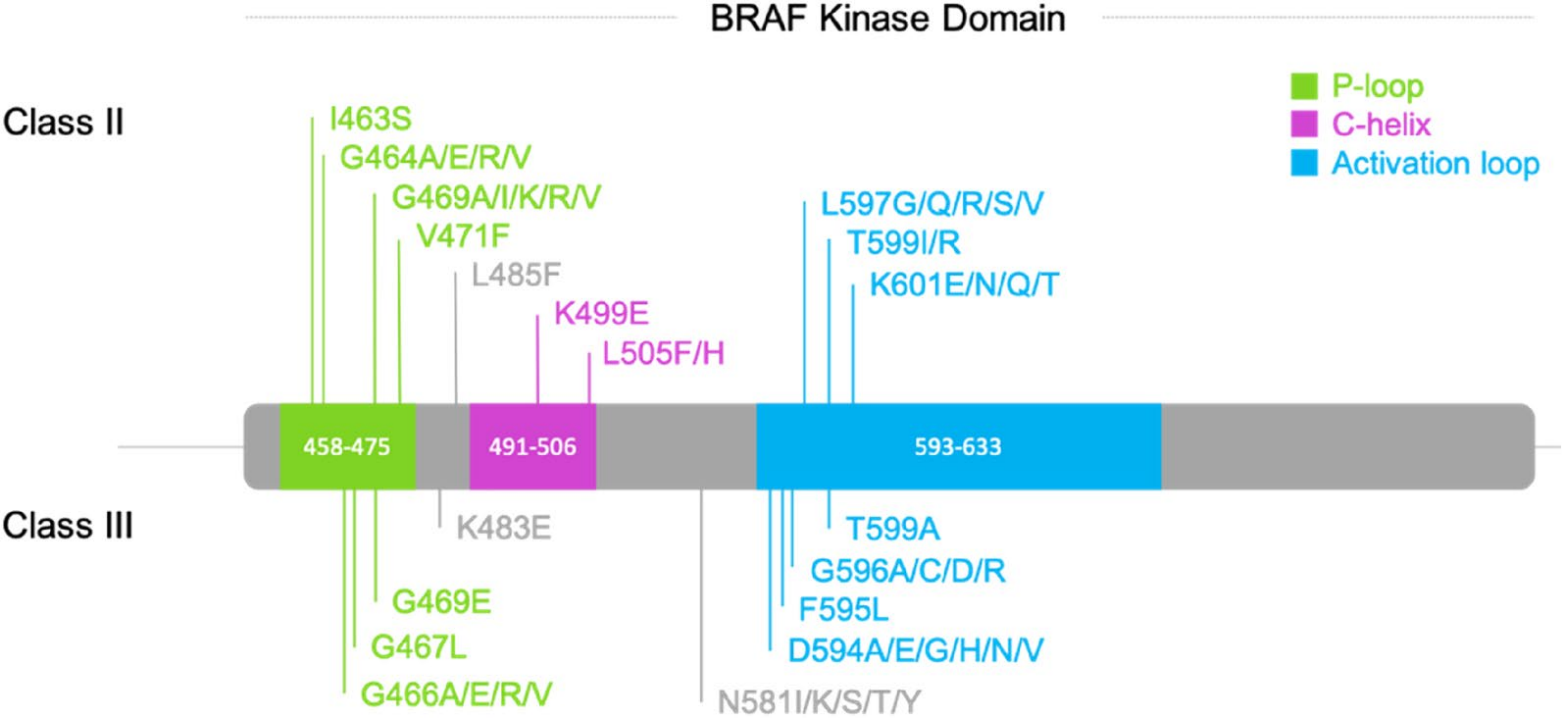
Position of the selected BRAF classes II and III mutations in a schematic representation of the BRAF kinase sequence. Crucial regions P-loop, C-helix and activation loop are colored in green, magenta and blue, respectively.

Mutations in the kinase domain can impact the protein structure and functionality, leading to pathogenic effects like a gain or decrease of function, which is crucial to know to propose an appropriate treatment to the individual patient in the context of personalized oncology. Three classes of BRAF mutations have been described based on their effects. Most of them are located in the kinase domain (Fig. 3). Class I involves only missense mutations of Val600. Classes II and III are more diverse as they comprise more positions and mutation types (missense, insertion, deletion and insertion-deletion, and gene fusions) and few positions can even lead to a class II or III depending on the variant (BRAF p.G469A is a class II mutation, while BRAF p.G469E is a class III one e.g.).^4,5^. At this day and based on the SPHN/SPO, ClinVar^6^, LOVD^7^ and JAX-clinical Knowledgebase^8^ databases, 373 BRAF mutations were reported, including 76 pathogenic ones (gain or loss of function). 52 of them are situated in the kinase domain. Classes II and III are centered on the active site, especially, around the chelating region of the phosphate tail with Mg^2+^. Of note, there are two exceptions to these observations: positions Glu549 and Glu586 (Fig. 3). These two outsiders probably participate in an unknown mechanism with another partner. We therefore removed them from our data set, which led to a total of 50 characterized mutations in the kinase domain: 26 for class II and 24 for class III (SI Table 1, Fig. 4).

**Figure 5:**
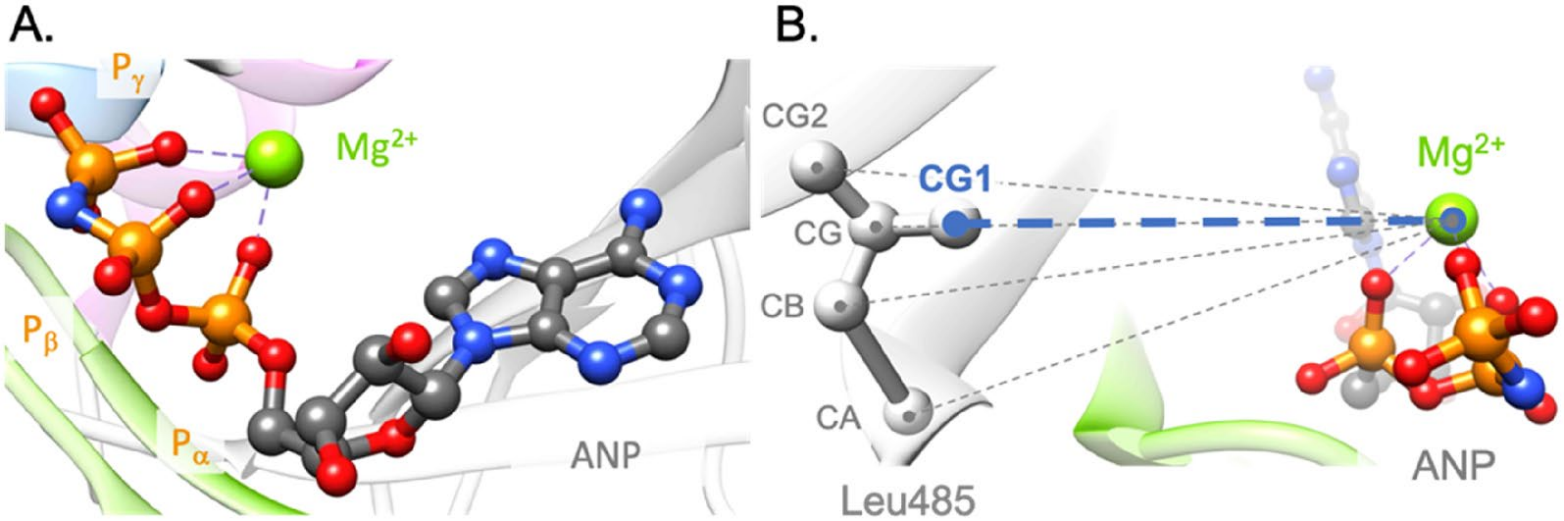
Key atoms and distances. (**A**) Representation of the key atoms selected for distance calculations, which are the Mg^2+^, and Pα, Pβ and Pγ from the ANP; (**B**) Example of the distance calculation between heavy atoms of Leu485 and Mg^2+^. The shortest distance, which will be used in our predictive model, is the one between atoms CG1 and Mg^2+^. ANP and Leu485 are represented in gray ball and stick, the Mg^2+^ in a green sphere and the protein in ribbons (pdb code: 6pp9) ^12^.

To build our predictive model, we made a selection of potential predictors, based on physicochemical and structural properties. We used the physicochemical features from the AAindex database, which gathers a lot of physicochemical and biochemical matrices representing the similarity of amino acids^9^. We retrieved and made a selection of 57 features based on specific criteria: size, volume, charge, hydrophobicity, hydrophilicity, polarity of a residue and its propensities to be buried or reside in a structured region (α-helix, β-sheet, coil) (SI Table 2). For each of these criteria, the corresponding features scales were normalized. To reduce the number of normalized features, we merged these data into two vectors of features per residue: one dedicated to the structural information (24 features), like for instance the propensity to be in a helix, and one for the other physicochemical parameters (33 features). Then, for each mutant, we calculated the city block similarity distance between the wild-type and the mutant residues, which reduces the number of features from 57 to 2. To complete the structural data, we focused on the 90 experimental 3D structures of the BRAF kinase publicly available from the PDB^10^ to extract relevant information. When a kinase mutant position is resolved in a structure, we: (i) checked whether it is in a structured region (α-helix, β-sheet, coil); ii) calculated the normalized B-factor as it reflects the flexibility of the position; (iii) calculated the solvent accessible surface area (SASA) of the side chains based on the CHARMM force field^11^. Of note, the two glycine hydrogens were designated as a side chain to consider glycine exposed to the solvent in the SASA calculation. Structural analysis of the classes II and III mutations show that they surround the phosphate tail binding area of the ligand. To take this finding into account, we selected 11 BRAF kinase structures co-crystallized with the Mg^2+^ and phosphoaminophosphonic acid-adenylate ester (ANP), an analog of the ATP (PDB codes: 6pp9^12^, 6v2w, 7m0t, 7m0u, 7m0v, 7m0w, 7m0x, 7m0y, 7m0z^13^). The aim of this selection is to stay close to the native active site conformation. As ATP would be immediately hydrolyzed in the kinase binding site, ANP is an ideal analog because an amine function in the phosphate tail prevents any catalytic reaction. Then, we selected key atoms: Mg^2+^, and the three phosphate atoms of the ligand (Pα, Pβ, Pγ) (Fig. 5). For each of the 7 BRAF kinase structures selected, we used FoldX software^14^ to build the mutant and wild-type structures, for which the wild-type residue is resolved in the corresponding structure. FoldX is an efficient software for predicting changes in free energy of folding upon mutations, whose predictive efficiency has been trained on an important set of mutations^15^. The predicted energetic perturbation (∆∆G_fold_) induced by the mutant is calculated and added to the feature set. For the next steps, we used both wild-type and mutant structures built by FoldX for the analysis. We preferred the new wild-type version of the initial one as FoldX repositioned the side chains of the residues around the positions of interest to remove potential constraints. For each mutant and wild-type position, the Euclidian distance between the coordinates of each heavy atom and the key selected ones (Mg^2+^ and ANP phosphorus atoms) is calculated and the shortest is kept (Fig. 5). Then, for each couple of distances calculated (ex: distances of wild-type *vs*. Mg^2+^ and mutant *vs*. Mg^2+^), we calculated the corresponding absolute difference, which leads to four values for each mutation (SI Table 3).

**Table 1:**
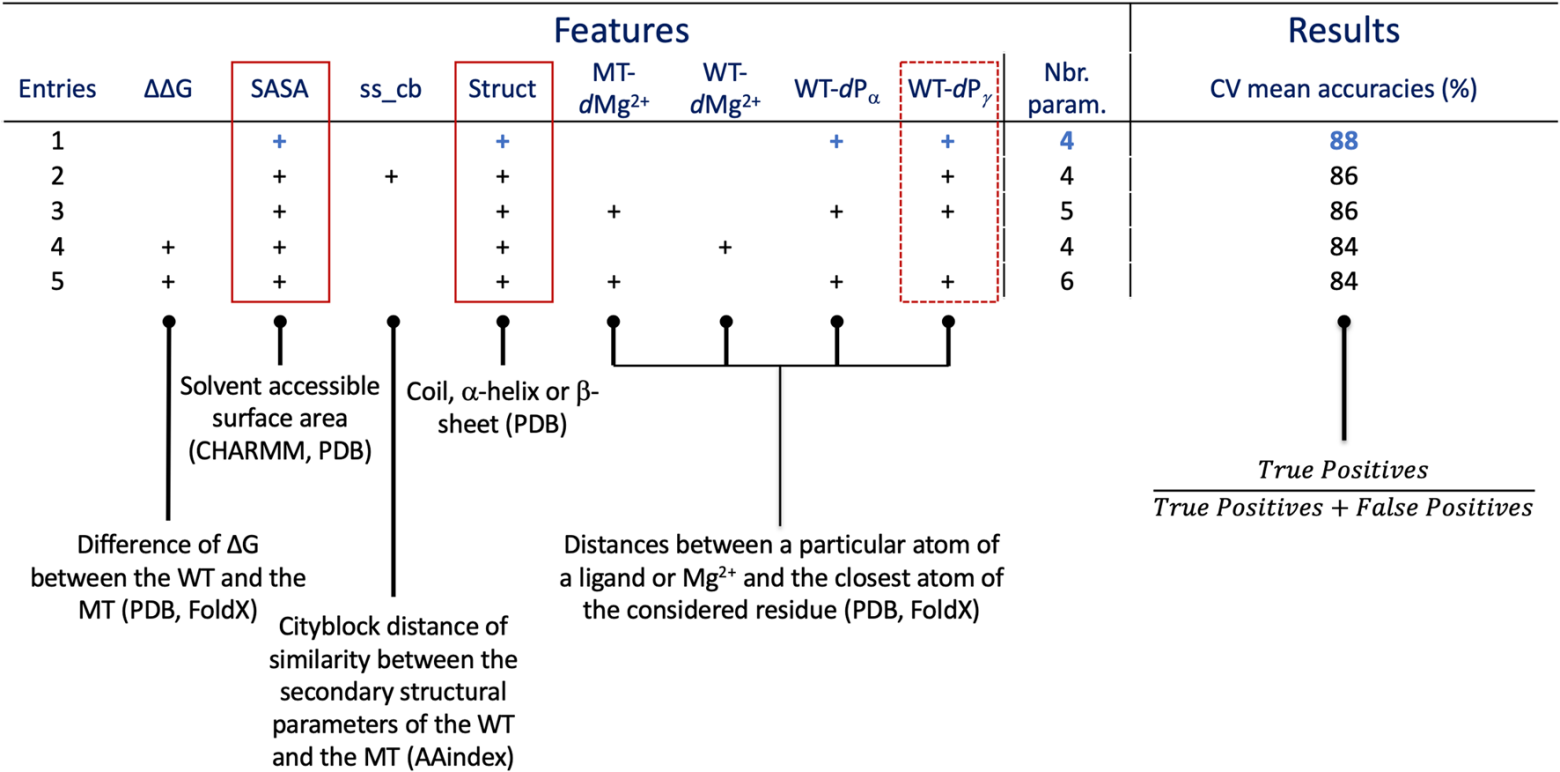
Best predictive models. Description of the parameters used and their corresponding accuracies. The databases or tools used to build the parameters are given in brackets. The red rectangles indicate the mainly used features.

*WT* wild-type, *MT* mutant, *CV* cross-validation.

**Table 2:** Functional parameters tested for the logistic regression approach model optimization.

*LogisticRegression* functional parameters	Options
*class_weight*	Balanced, None
*solver*	newton-cg, lbfgs, sag, saga
*max_iter*	1000, 5000, 10,000

**Figure 6:**
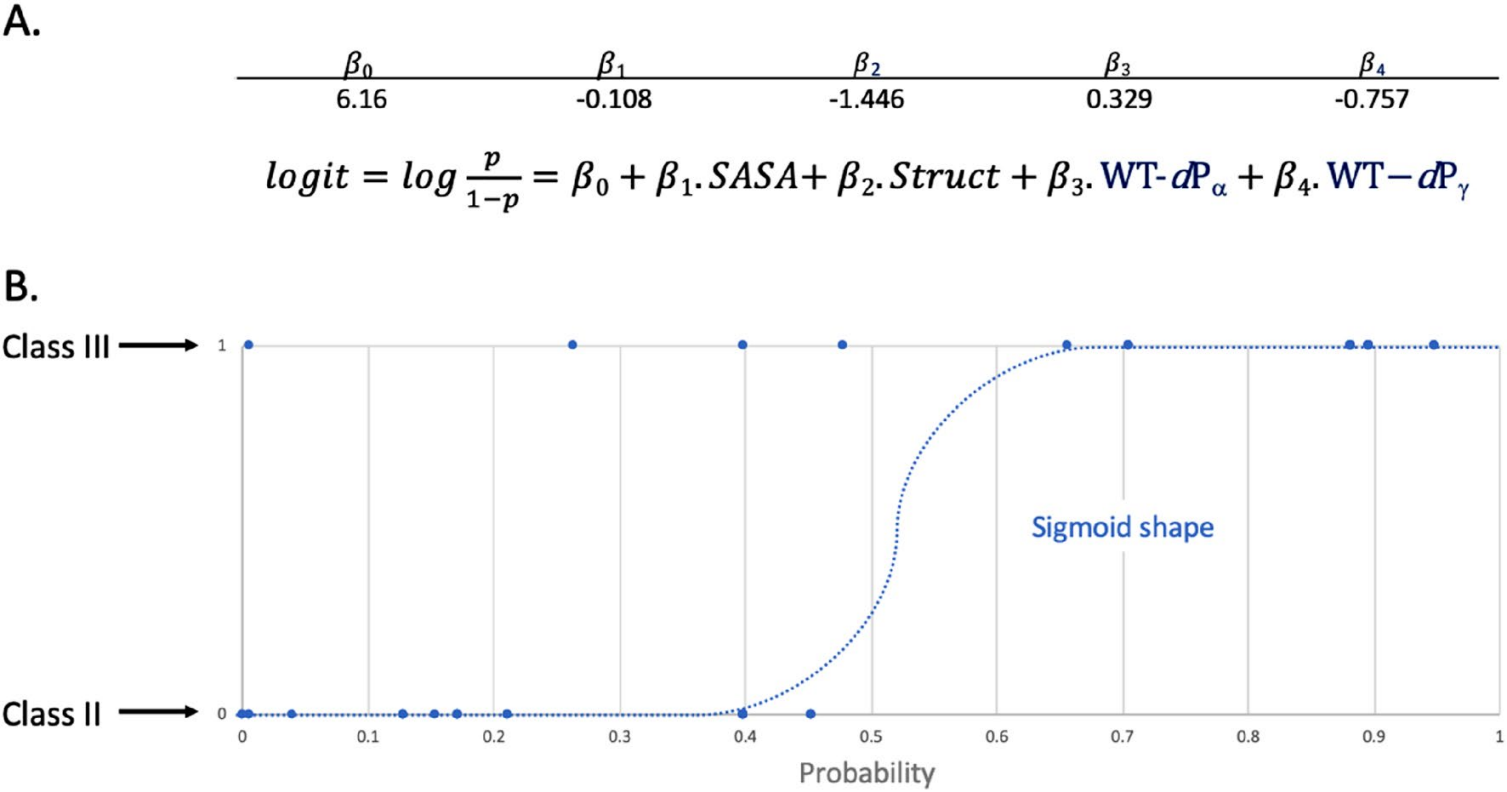
Logistic regression model results. (**A**) Coefficients obtained for the best predictive model and the logistic regression equation of the model; (**B**) Of note, several points superimpose themselves which gives the impression of having fewer points.

We ended with a total of 18 features, 16 extracted from the experimental structures (listed in SI Table 3) and 2 condensed ones from the physicochemical data, for 50 mutations. Such a ratio between data points and features could lead to overfitted machine learning models that could compromise the quality of the results^16^. As we cannot increase the data set size to overcome this problem, we concentrated on simple predictive models, reducing the number of used features. Different algorithms were tested, like k-nearest neighbors, multiple layer perceptron, support vector machines, decision trees and logistic regression. After testing and analyzing results, the logistic regression model was found to provide the best compromise between predictive ability and low likelihood of being overfitted (data not shown). Indeed, as the output of the model (class II or class III) can be encoded as a binary number, logistic regression is an approach of choice for this kind of prediction exercise. We tried several combinations from the list of 18 features generated and reduced their number while optimizing the accuracy of our model and analyzing their corresponding coefficients (SI Table 4). We started by modifying specific functional parameters, like the solver (newton-cg, lbfgs, sag, saga), the maximum of iteration limit (1000, 5000, 10,000) and the weight class option (None, balanced), all while testing the different physicochemical and structural features (Table 2). The maximum number of iterations required to converge highly depends on the solver used. For example, the feature combination mentioned in Table 1, entry 1 converges in 17 steps when using newton-cg as a solver whereas it requires 4882 steps with saga one. Consequently, we choose to use high limits for the maximum number of iterations to ensure that models always converge properly. We noticed that the physicochemical feature can be removed as it does not improve the accuracy of the models tested and the coefficient values were very close to zero compared to other ones, whereas structural based features seem to have an important role in the efficiency of the tested models. We ended with a selection of five models, which show the highest accuracy of 90% with the newton-cg solver, a maximum of iterations of 1000 and no weight class option. We ran the process 1000 times with their optimized parameters, plus 10 cross-validation (CV) steps. The means of the accuracy score resulting from the training and the CV steps were calculated and used to rank the different models. The overall execution process is described in the “Material and Methods” section. A mean accuracy of 90% was obtained for the five models. Mean CV scores were comprised between 84 and 88% (Table 1, entries 1–5). The best selected features only include structural ones: mutant energetic perturbations (∆∆Gfold), SASA, distances between the Mg^2+^ and the wild-type and mutated residues, and distances between the wild-type residues and the ANP Pα and Pγ. The best model presents a mean CV accuracy of 88% with the following parameters (Table 1, entry 1): SASA, structural environment (struct), the distances between the wild-type residue and the atoms Pα and Pγ of the ANP (WT-dPα and WT-dPγ, respectively). The logistic coefficients of this predictive model are presented in Fig. 6A with the corresponding equation involving the selected parameters. As expected, the plot of predictive results shows a sigmoid shape (Fig. 6B). Most of the data points are found on the plateaus of the sigmoid curve, illustrating the quality of the prediction. Of note, some of the 50 data points are superimposed on this graph, which hides some of them.

**Figure 7:**
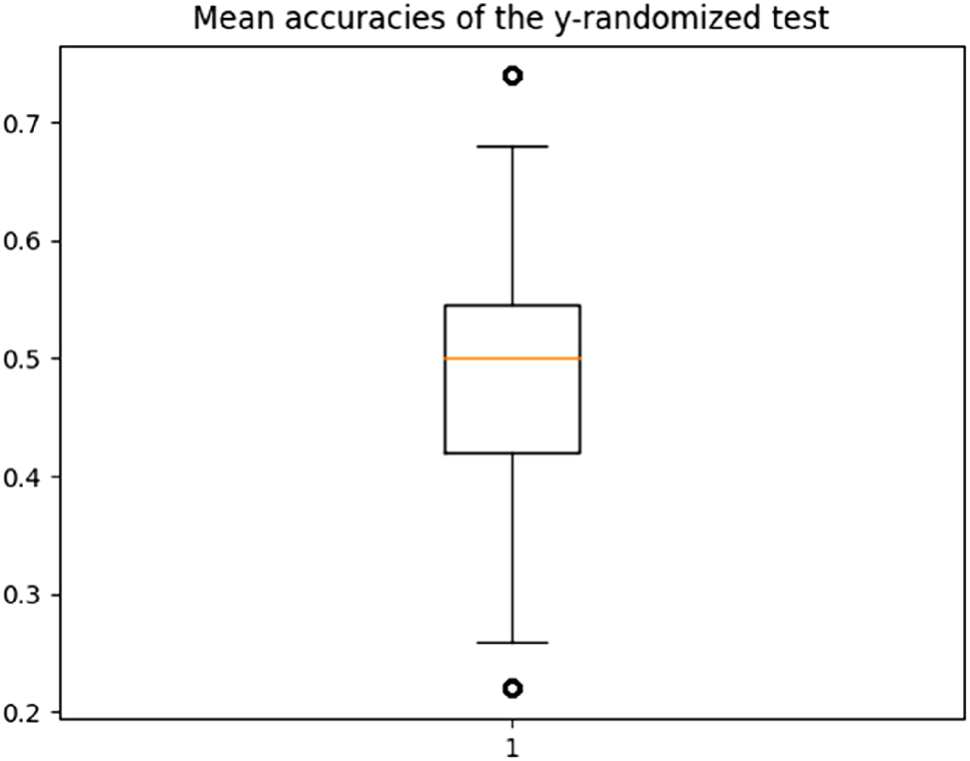
Randomized process results. Mean accuracy boxplot resulting from the accurracies obtained by the y-randomized process.

To ensure the reliability of our model we tested it on randomized data, performing a so-called y-randomization process, where the real experimental outcome is systematically replaced by a new random one. To perform this, we ran 100 times the overall process using the selected features from the final model on an y-randomized dataset (i.e., the class attributions is randomized). A mean accuracy of 48% was obtained. No value reached the one of the real model, as the randomized accuracy values range from 22 to 74% (Fig. 7). These results confirm the reliability of our model.

## Materials and methods

Known mutations and their corresponding classes were retrieved from the literature^4–8^. All scripts were written in Python3.

For this study, we created a list of 73 features. 57 of them are physiochemical and structural properties, retrieved from the AAindex database, that were classified in two categories: the structural and physicochemical properties which are respectively composed of 24 and 33 features (for more details, please see SI Table 2). The range of values was normalized for each feature. Then, we created two vectors of features, one containing the 24 structural features and another one the 33 physicochemical features. The 16 features based on structural analysis were obtained from BRAF experimental X-ray structures retrieved from the PDB. To have data from structures that have a binding site environment close to the one in the presence of the natural substrate ATP, we selected 11 BRAF structures co-crystallized in presence of Mg^2+^ and ANP, which is a non-hydrolyzable analog of ATP (PDB codes: 6pp9^12^, 6v2w, 7m0t, 7m0u, 7m0v, 7m0w, 7m0x, 7m0y, 7m0z^13^). The phosphate atoms of the phosphate tail of ANP are mentioned as Pα, Pβ and Pγ.

Scipy, which is a python library, was used for similarity distance calculations^18^. CHARMM 36 all hydrogen force field was used for the SASA calculations with the top_all22 and par_all22 files, for topology and parameter files respectively^11^. FoldX version 5 was used with the command line PositionScan to generate the structures and to calculate the predictive energetic perturbation of the mutant compared to the wild-type structure^14^. It starts with the mutation of the desired residue into alanine and detects neighbors before mutating it again into itself. The same steps are applied to the selected neighbors which allows a side chain optimization around the position of interest and to build the wild-type reference structure. Then, this latter is mutated into the target, the mutant structure is built and the energetic predictive perturbation is calculated (∆∆G = ∆G_mutant_ − ∆G_wild-type_) in kcal/mol. FoldX generated two models: one of the wild-type and one of the mutant in which it rearranged the side chain positions. To stay coherent with the predictive mutant models, we used the wild-type structures generated by FoldX v.5 for the Euclidean distance’s measures.

Scikit-learn Python was used for the machine learning steps. It integrates a wide range of machine learning algorithms for medium scale supervised and unsupervised tasks^17^. From the sklearn.linear_model, we imported the LogisticRegression module for the logistic regression approach. To select the most appropriate models, several functional parameters were tested using the GridSearchCV module imported from sklearn.model_selection which allows an exhaustive search over specified parameter values. We chose to tune classical parameters like the class_weight, the solver which is the algorithm used for the optimization and the max_iter for the maximum number of iterations taken for the solver to converge (Table 2).

The complete data set was randomly split in training and test sets in the respective following proportions of 80% and 20%. The models were trained on the training set and tested on the testing set. Of note, because of a low amount of data available, we decided to use them all to create the training and testing sets, without generating an external one. To face this limitation, the training process to get the models was built to test their robustness to ensure the quality of our results. A first accuracy score was calculated on the given test data and labels. When this score was higher than 80%, the corresponding models were kept and 10 CV steps were made. Then the mean CV score was calculated and compared to the previous accuracy score, to verify that no drop in accuracy was observed in CV. This process was run 1000 times and the averages of the performed accuracies, with and without CV, were calculated, like the average of the parameter coefficients of the logistic regression equation. Table 1 presents the top five models obtained using this workflow.

To perform and test the y-randomization process, the labels (i.e., class information) were randomized using the Python module *random*. Then, based on the best features obtained from the best model, the overall process was ran 100 times. The mean accuracies obtained were plot into a boxplot using Python matplotlib library.

## Conclusions

BRAF is a well-known onco-driver gene of the MAP kinase signaling pathway. Non-synonymous mutations within the BRAF gene can be classified as class I, II or III depending on their effect on the BRAF kinase activity. Most of the mutations occur in the kinase domain and so far, class I mutations exclusively involved missense mutations of Val600. We focused on the known missense mutations of classes II and III detected in the kinase domain to develop a tool that allows the prediction of the potential class of missense mutations detected in the BRAF kinase domain. Using a logistic model trained with a wise parameter and feature selection and known mutation class data retrieved from the literature, we developed an efficient and robust model capable of predicting with high mean accuracy scores of 90% and 88%, based on the validation set and cross-validating steps respectively, if a suspicious missense mutation impacting a position other than Val600 could be a class II or III. Because of the limited experimental data regarding mutation class available, we could not test our model on an external data set, that would have not been used during the training of our models. However, the overall process was built to minimize as much as possible any overfitting effects. In the future, we hope that new experimentally determined BRAF mutations of known classes will become available to test our model externally.

As comprehensive genomic profiling of patients’ tumor samples is increasingly being used in clinical routine, it is likely that more uncharacterized BRAF gene mutations will be detected in the future. A definite assignment to a given mutational class will involve biochemical testing of dimerization of RAF molecules, the MAPK signaling output and possibly also how these can be influenced by application of tyrosine kinase inhibitors of BRAF and MEK. Those are extensive in vitro experiments, which in clinical routine are often times not feasible, particularly not at a turnaround time, which would be necessary to support clinical decision-making regarding therapeutic options. Our prediction algorithm will therefore provide a valuable tool to bioinformaticians, molecular pathologists and clinicians to get an initial idea of the potential biochemical class of the detected mutation and to issue a therapy suggestion for patients presenting with new uncharacterized BRAF gene mutations in their tumor specimens.

## Supplementary Information

Supplementary Information.
