# Supplementary material for: Structure-based prediction of BRAF mutation classes using machine-learning approaches
Source: Sci Rep. 2022 Jul 22;12:12528. doi: 10.1038/s41598-022-16556-x (PMC9307832; doi:10.1038/s41598-022-16556-x)
Supplement: Supplementary file 1 — Supplementary Information. [file 41598_2022_16556_MOESM1_ESM.docx]

Structure-based prediction of BRAF mutation classes using machine-learning approaches

Fanny S. Krebs^1^, Christian Britschgi^4^, Sylvain Pradervand^3^, Rita Achermann^5^, Petros Tsantoulis^6^, Simon Haefliger^7^, Andreas Wicki^4^, Olivier Michielin^2,3^ and Vincent Zoete^1,2,*^

^1^ Computer-Aided Molecular Engineering, Department of Fundamental Oncology, University of Lausanne, Epalinges, Switzerland

^2^ Molecular Modelling group, Swiss Institute of Bioinformatics, Lausanne, Switzerland

^3^ Center for Precision Oncology, Department of Oncology, Centre Hospitalier Universitaire Vaudois, University of Lausanne, Lausanne, Switzerland

^4^ Department of Medical Oncology and Hematology, University Hospital Zurich, Comprehensive Cancer Center Zurich, University of Zurich, Zurich, Switzerland

^5^ Department of Radiology, Clinic of Radiology & Nuclear Medicine, University Hospital Basel, University of Basel, Basel, Switzerland

^6^ Department of Oncology, Hôpitaux Universitaires de Genève, University of Geneva, Geneva, Switzerland

^7^ Department of Medical Oncology, Inselspital, Bern University Hospital, University of Bern, Bern, Switzerland

***** Correspondence: Vincent.zoete@unil.ch

**Supplementary data**

SI Table 1: List of the classes II and III BRAF mutations localized in the kinase domain and selected in this study

| **Class II** | **Class III** |
| --- | --- |
| I463S | G466A |
| G464A | G466E |
| G454E | G466R |
| G454R | G466V |
| G454V | S467L |
| G469A | G469E |
| G469I | K483E |
| G469K | N581I |
| G469R | N581K |
| G469V | N581S |
| V471F | N581T |
| L485F | N581Y |
| K499E | D594A |
| L505F | D594E |
| L505H | D594G |
| L597G | D594H |
| L597Q | D594N |
| L597R | D594V |
| L597S | F595L |
| L597V | G596A |
| T599I | G596C |
| T599R | G596D |
| K601E | G596R |
| K601N | T599A |
| K601Q |  |
| K601T |  |

SI Table 2: AAindex codes of the features selected [Kawashima, S. and Kanehisa, M. AAindex: Amino Acid Index Database. NAR **2000**, 28, 374]

| **Type of parameters** | **Parameter codes from AAindex Database** |
| --- | --- |
| Burial amino acid | ZHOH040103, CHOC750101, WERD780101 |
| Size/Volume | PONJ960101, HARY940101, FAUJ880103, KRIW790103 |
| Charge | FAUJ880111, FAUJ880112, KLEP840101 |
| Hydrophobicity | ARGP820101, CIDH920103, CIDH920105, EISD840101, GOLD730101, JOND750101, LEVM760101, MANP780101, PRAM900101, SWER830101, ZIMJ680101, PONP930101, WOLR790101, KIDA850101, BLAS910101, ENGD860101, FASG890101 |
| Hydrophilicity | ROBB790101, WOLR810101, HOPT810101, KUHL950101 |
| Polarity | CHAM820101, GRAR740102 |
| Secondary structure | CHAM830101, FINA770101, PTIO830101, ISOY800108, NAGK730103, TANS770109, ROBB760112, BURA740101, CHOP780201, LEVM780101, LEVM780104, MAXF760101, NAGK730101, PALJ810101, PALJ810102, PALJ810108, TANS770101, CHOP780202, CRAJ730102, LEVM780102, LEVM780105, PALJ810103, PALJ810104, PALJ810111 |

SI Table 3: Features generated based on experimental structures analysis per known mutated and resolved position

| **Features based on experimental structures** |
| --- |
| Surface Accessible to the Solvent Area (Calculated using the CHARMM force field) |
| Structural information (coil, α-helix, β-sheet) |
| Normalized B-factor |
| Predicted energetic perturbation (∆∆Gfold, calculated using FoldX) |
| Wild-type vs. Mg^2+^ distance (WT-*d*Mg^2+^) |
| Wild-type vs. P_α_ distance (WT-*d*P_α_) |
| Wild-type vs. P_β_ distance (WT-*d*P_β_) |
| Wild-type vs. P_γ_ distance (WT-*d*P_γ_) |
| Mutant vs. Mg^2+^ distance (MT-*d*Mg^2+^) |
| Mutant vs. P_α_ distance (MT-*d*P_α_) |
| Mutant vs. P_β_ distance (MT-*d*P_β_) |
| Mutant vs. P_γ_ distance (MT-*d*P_γ_) |
| abs\| (Wild-type vs. Mg^2+^ distance) – (Mutant vs. Mg^2+^ distance)\| |
| abs\| (Wild-type vs. P_α_ distance) – (Mutant vs. P_α_ distance)\| |
| abs\| (Wild-type vs. P_β_ distance) – (Mutant vs. P_β_ distance)\| |
| abs\| (Wild-type vs. P_γ_ distance) – (Mutant vs. P_γ_ distance)\| |

SI Table 4: Twenty best logistic regression models results. Description of the features used and their corresponding mean cross-validation scores (See material and methods section for more information). WT, MT and CV stand for wild-type, mutant and cross-validation steps, respectively. The solver used was newton-cg, a maximum of iteration of 1000 was set and no weight class option was selected.

| **Entries** | **∆∆G** | **sasa** | **ss_cb** | **struct** | **MT-*d*Mg^2+^** | **WT-*d*Mg^2+^** | **MT-*d*P_g_** | **WT-dP_a_** | **WT-*d*P_g_** | **Nbr. param.** | **CV mean score (%)** |
| --- | --- | --- | --- | --- | --- | --- | --- | --- | --- | --- | --- |
| 1 |  | + |  | + |  |  |  | + | + | 4 | 88 |
| 2 |  | + | + | + |  |  |  |  | + | 4 | 86 |
| 3 |  | + |  | + | + |  |  | + | + | 5 | 86 |
| 4 | + | + |  | + |  | + |  |  |  | 4 | 84 |
| 5 | + | + |  | + | + |  |  | + | + | 6 | 84 |
| 6 | + | + |  | + |  |  |  | + | + | 5 | 82 |
| 7 | + | + |  | + | + |  |  | + | + | 6 | 82 |
| 8 | + | + |  | + |  |  |  | + | + | 5 | 82 |
| 9 | + | + |  | + | + |  |  | + |  | 5 | 82 |
| 10 |  | + | + | + | + |  |  | + | + | 6 | 80 |
| 11 | + | + |  | + | + |  | + | + | + | 7 | 80 |
| 12 | + | + | + | + | + |  | + | + |  | 7 | 80 |
| 13 |  | + | + | + | + |  | + | + | + | 7 | 80 |
| 14 |  | + | + | + | + | + |  |  | + | 6 | 78 |
| 15 | + | + | + |  | + | + |  | + | + | 7 | 78 |
| 16 |  | + |  | + |  |  |  | + | + | 4 | 76 |
| 17 |  | + |  | + | + |  |  | + | + | 5 | 76 |
| 18 |  | + | + | + |  |  |  |  |  | 3 | 72 |
| 19 |  |  |  |  | + | + |  |  | + | 3 | 66 |
| 20 | + | + | + |  | + |  |  |  |  | 4 | 62 |
